# Prognostic value of myopic disk deformation in myopic choroidal neovascularization: A 6-year follow-up study

**DOI:** 10.3389/fmed.2022.947632

**Published:** 2022-08-01

**Authors:** Ye Eun Han, Yoon Jeon Kim, Hyun Seung Yang, Byung Gill Moon, Joo Yong Lee, June-Gone Kim, Young Hee Yoon

**Affiliations:** ^1^Department of Ophthalmology, Asan Medical Center, University of Ulsan College of Medicine, Seoul, South Korea; ^2^Department of Ophthalmology, Seoul Shinsegae Eye Center, Eui Jung Bu, South Korea

**Keywords:** myopic choroidal neovascularization, myopic disc deformation, β-zone peripapillary atrophy, optic disc tilt, degenerative myopia

## Abstract

**Purpose:**

To evaluate the clinical characteristics of myopic choroidal neovascularization (mCNV) according to peripapillary atrophy (PPA) and optic disk tilt and to explore whether those myopic disk deformations are associated with the prognosis of mCNV.

**Methods:**

Patients with subfoveal mCNV who received intravitreal bevacizumab injection and followed for ≥3 years were included. PPA was quantified as area of the ß-zone PPA/disk area ratio (PDR) and optic disk tilt as the tilt ratio (the longest/shortest disk diameter). We compared the clinical characteristics in terms of PDR and tilt ratio and identified the poor prognostic factors using Logistic regression and Cox proportional hazard model.

**Results:**

Among 80 eyes of 80 patients, 29 (36.30%) eyes developed macular atrophy during 80.71 ± 34.76 months. PDR and tilt ratio are strongly correlated with each other (*P* = 0.004). Higher PDR showed significant correlations with longer axial length (*P* = 0.013), worse baseline and final VA (*P* = 0.007 and *P* = 0.047), and thinner subfoveal choroidal thickness (*P* = 0.039), while higher tilt ratio showed significant correlations only with longer axial length (*P* = 0.036). High PDR was also an independent risk factor for both macular atrophy (OR = 2.257, *P* < 0.001) and poor visual outcome (HR = 1.174, *P* = 0.007), while high disk tilt ratio was not.

**Conclusion:**

Subfoveal mCNV with higher ß-zone PPA area/disk area ratio had worse functional and structural outcomes.

## Introduction

Myopic choroidal neovascularization (CNV) is a vision-threatening complication (1) of degenerative myopia, and develops in at least 5–11% of patients with a high degree of myopia (2). The progressive and excessive elongation of the eyeball causes various degenerative changes involving the sclera, choroid, and retina, and many cases with myopic CNV develop macular atrophies and scarring that lead to legal blindness (3, 4). In previous studies, old age, long axial length, large CNV size, subfoveal location of the CNV, thin subfoveal choroidal thickness, and poor baseline best-corrected visual acuity (BCVA) were identified as poor prognostic factors for myopic CNV (5–10). The exact mechanism for the development and progression of myopic CNV is unknown, but a combination of mechanical and hemodynamic stress with genetic predisposition is a likely culprit (5). In myopia, morphological changes of the optic disk such as formation of peripapillary atrophy (PPA) and optic disk tilt occur (11, 12). PPA formation and disk tilting are known to be accompanied by mechanical scleral stretching and posterior deformation secondary to axial elongation (13, 14). In addition, a recent study (15) found that retinal microvasculature of macular and peripapillary regions was significantly reduced according to the degree of optic nerve head deformations in highly myopic eyes.

Based on these findings, we hypothesized that the myopic optic disk deformations and prognosis of myopic CNV are associated with each other, considering that they share the common mechanical and hemodynamic mechanisms in their pathogenesis. Therefore, in this study, we aimed to evaluate the clinical characteristics of myopic CNV based on PPA and optic disk tilt and to explore whether these myopic disk deformations were associated with the prognosis of myopic CNV.

## Materials and methods

### Study patients

This was a retrospective observational study conducted at Asan Medical Center, a tertiary referral center in Seoul, South Korea. We reviewed the medical records of all consecutive treatment-naïve patients treated with intravitreal bevacizumab injections after a preliminary diagnosis of subfoveal myopic CNV and who were regularly followed for more than 3 years between January 2007 and September 2020. The criteria for exclusion were as follows: history of intraocular surgeries other than cataract surgery; history of intraocular treatments (e.g., photodynamic therapy, laser photocoagulation, and intravitreal injection) due to other ocular pathologies; history of ocular trauma, inflammation, or infection; and media opacities that could affect visual acuity. All procedures were conducted in accordance with the tenets of the Declaration of Helsinki, and the study design was approved by the Institutional Review Board (IRB) of Asan Medical Center (Seoul, South Korea; IRB No. 2020-1421). Due to the retrospective design of the study and the use of de-identified patient data, the IRB of Asan Medical Center waived the need for written informed consent from patients.

### Ocular evaluation and treatment

At the initial and following visits to the clinic, all patients underwent a complete ophthalmologic evaluation, including a review of the ophthalmologic history, measurement of the BCVA and axial length by IOL Master 500 (Carl Zeiss Meditec AG, Gena, Germany), slit lamp biomicroscopy, spectral domain optical coherence tomography (SD-OCT, Heidelberg Engineering, Heidelberg, Germany), including extended depth image, fundus autofluorescence (FAF; HRA-2; Heidelberg Engineering, Heidelberg, Germany), fluorescein angiography (FA; Topcon TRC-50DX, Topcon, Tokyo, Japan), and indocyanine green angiography (ICGA; HRA-2; Heidelberg Engineering, Heidelberg, Germany), and funduscopic examinations through dilated pupils by retinal specialists.

Macular status, including the CNV, intra-/sub-retinal fluid, lacquer crack, subfoveal choroidal thickness, and macular atrophy, was assessed using SD-OCT, FA, ICGA, and FAF. The presence of CNV was defined by the growth and invasion of new choroidal vessels through Bruch’s membrane on SD-OCT. To exclude CNVs caused by other diseases besides pathologic myopia, we diagnosed myopic CNV only when they have a solid relationship to myopic degenerative changes (i.e., posterior staphyloma, Fuch’s spots, lacquer crack, and peripapillary intrachoroidal cavitation) without signs for other ocular diseases throughout the whole cohort. Especially because distinguishing the inflammatory lesions (i.e., punctate inner choroidopathy and multifocal choroiditis) and myopic CNV is challenging, we excluded the eyes with inflammatory signs (i.e., late staining of the optic disk margin in FA and late hypofluorescent dots in ICGA). Subfoveal myopic CNV was defined as any portion of the CNV that developed in degenerative myopia involving the foveal center. The size of the CNV lesion and the subfoveal choroidal thickness (between the Bruch membrane and the inner portion of the sclera) were measured manually (8). For these measurements, the Heidelberg Eye Explorer (v 1.10.4.0, Heidelberg Engineering) was used with the HRA/Spectralis Viewing Module (v 6.16.7.0) and the HSF Region Finder (v 2.6.5.0) using AutoRescan images. Two independent examiners (YEH and YJK) conducted all ocular evaluations and measurements, and their data were averaged for the final analysis. The intraclass correlation coefficient (ICC) was used to examine the interobserver agreement. The ICC value was 0.950 for CNV size and 0.930 for the choroidal thickness. Macular atrophy was defined as the area of hypofluorescence in FAF with defective retinal pigment epithelium in SD-OCT. Degenerative myopia was defined as eyes with a spherical equivalent refractive error of more than −8.0 diopters or an axial length longer than 26.5 mm, accompanied by posterior chorioretinal degenerative changes.

All patients followed the “1 + PRN” protocol to resolve CNV activity. Active CNV was determined if leakage on FA/ICGA or intra-/sub-retinal fluid or fuzzy margin of CNV was observed on SD-OCT. If CNV activity was resolved on the subsequent follow-up OCT after one injection, no further injection was given until recurrences were observed. However, if CNV activity remained, additional injections were given until CNV was stabilized. Recurrence of myopic CNV was defined as the reactivation of CNV activity at least 3 months after the initial resolution.

### Optic disk feature measurements

**FIGURE 1:**
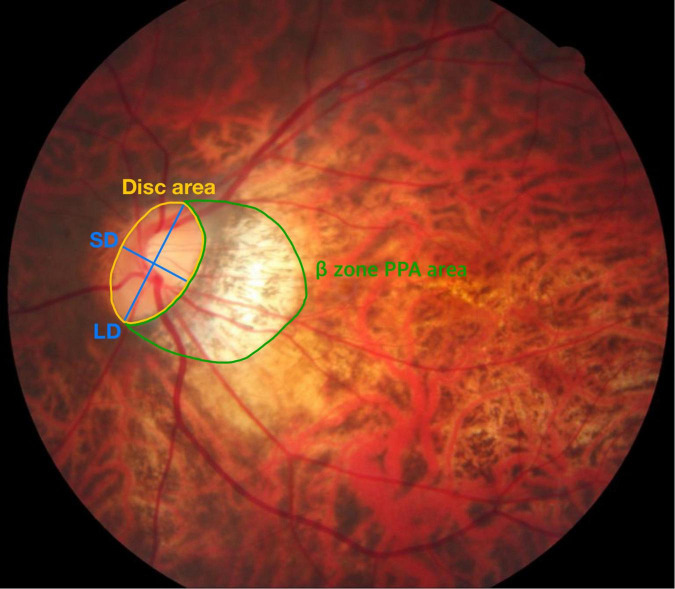
Measurement of the β-PPA-to-disc area ratio (PDR) and tilt ratio. The optic disc area (demarcated by the yellow line) and β-zone peripapillary area (β-PPA, demarcated by the green line) were automatically calculated in pixel area using the Image J software. The β-PPA-to-disc area ratio (PDR) was used to minimize the effects of photographic magnification error and represent the β-PPA. Blue lines indicate the shortest diameter (SD) and the longest diameter (LD) of the optic disc. The tilt ratio was defined as the LD-to-SD ratio.

ß-zone peripapillary atrophy (ß-PPA) and optic disk tilt were analyzed from color fundus photography (TRC-50XD, Topcon Corp, Tokyo, Japan) using the ImageJ software (version 1.52; Wayne Rasband, National Institutes of Health, Bethesda, MD, United States; Figure 1). To minimize the effects of photographic magnification error and represent the size of the ß-PPA, we calculated the ß-PPA-to-disk area ratio (PDR; 16). The margin of the optic disk was defined as the inner border of Elschnig’s sclera ring, and ß-PPA was defined as the inner crescent of chorioretinal atrophy with visible sclera and choroidal vessels. Optic disk tilt was quantified by the tilt ratio (the longest to shortest disk diameter). An optic disk was classified as a tilted disk when the tilt ratio was greater than 1.30 (17). All demarcations and measurements were manually performed and averaged data from two independent examiners (YEH and YJK) was used in the final analysis. The ICC value for PDR was 0.964 (95% CI, 0.943 to 0.997) and disk tilt ratio was 0.970 (95% CI, 0.953–0.981).

### Statistical analyses

The following variables were analyzed in each patient: (i) demographic variables (i.e., age, sex, and systemic diseases), (ii) baseline ocular characteristics (i.e., lens status, axial length, refractive errors, presence of lacquer crack, CNV size, subfoveal choroidal thickness, disc parameters, and BCVA), and (iii) treatment outcomes (i.e., number and timing of anti-VEGF injections, recurrence of CNV, occurrence of macular atrophy, and final BCVA).

Descriptive statistics of the baseline characteristics are presented in numbers and percentages for categorical variables and mean ± standard deviation for continuous variables. Student’s *t*-test was used to compare continuous variables. Partial correlation analysis adjusted for age and sex was used to investigate the association between myopic disk features (the ß-PPA and disk tilt) with baseline ocular characteristics and treatment outcomes. Logistic regression and Cox proportional hazards model were used to identify factors associated with the occurrence of macular atrophy at the last follow up visit and poor visual prognosis (<20/60, Snellen mean), respectively. Univariate analyses were performed for each variable, and those with *P* values < 0.1 were included in the multivariable analysis with a forward elimination process. All statistical analyses were performed using the IBM SPSS Statistics for Windows, Version 21.0 (IBM Corp., Armonk, NY, United States).

## Results

A total of 80 eyes of 80 patients with subfoveal myopic CNV were included in the study. The baseline demographics, ocular characteristics and treatment outcomes are summarized in Table 1. During 80.71 ± 34.76 months (range: 36.0–157 months) of follow-up, the patients received 5.52 ± 4.75 injections. The mean LogMAR BCVA was changed from 0.64 ± 0.54 to 0.75 ± 0.70. Twenty-seven (33.80%) eyes experienced mCNV recurrence and 29 (36.30%) eyes developed macular atrophy.

**TABLE 1:** Baseline demographics and ocular characteristics of the patients in this study and their treatment outcomes at the last visit.

Demographics	80 eyes of 80 patients
Age, years	51.89 ± 16.03
Female, *N* (%)	49 (61.30)
Diabetes mellitus, *N* (%)	5 (6.30)
Hypertension, *N* (%)	19 (23.80)
**Ocular characteristics**	
Lens status: pseudophakia, *N* (%)	39 (48.70)
Axial length, mm	29.86 ± 1.76
Refractive errors, diopters	−11.98 ± 5.29
Presence of lacquer crack, *N* (%)	24 (30.00)
CNV size, mm^2^	0.77 ± 0.64
sfCT, μm	97.17 ± 85.44
Baseline BCVA, LogMAR	0.64 ± 0.54
**Optic disk features**	
Disk tilt ratio	1.42 ± 0.27
ß-zone PPA area/disk area	3.28 ± 3.03
**Treatment outcomes**	
Total follow-up duration, months	80.71 ± 34.76
Total No. of anti-VEGF injections	5.52 ± 4.75
Recurrence of CNV, *N* (%)	27 (33.80)
BCVA at the last visit, LogMAR	0.75 ± 0.70
BCVA changes during follow-up, LogMAR	0.10 ± 0.75
Macular atrophy at the last visit	29 (36.30)

CNV, choroidal neovascularization; sfCT, subfoveal choroidal thickness; BCVA, best corrected visual acuity; PPA, peripapillary atrophy; and VEGF, vascular endothelial growth factor.

Partial correlation coefficient between myopic optic disk deformations (PDR and tilt ratio) with baseline ocular characteristics and treatment outcomes adjusted for age and sex are shown in Table 2. There was a strong correlation between the PDR and tilt ratio (*r* = 0.692, *P* = 0.004). PDR showed significant correlations with axial length (*r* = 0.621, *P* = 0.013), baseline and final LogMAR BCVA (*r* = 0.661, *P* = 0.007 and *r* = 0.520, *P* = 0.047, respectively), and subfoveal choroidal thickness (*r* = −0.537, *P* = 0.039), while the tilt ratio showed significant correlations only with axial length (*r* = 0.544, *P* = 0.036).

**TABLE 2:** Correlation between optic disk features and baseline ocular characteristics and treatment outcomes.

	Disk tilt ratio		ß-zone PPA area/disk area	
	*r*	*P*-value	*r*	*P*-value
**Baseline ocular characteristics**				
Axial length	0.544	0.036*	0.621	0.013*
Baseline BCVA	0.373	0.170	0.661	0.007*
CNV size	0.001	0.997	0.118	0.674
Subfoveal choroidal thickness	−0.421	0.118	−0.537	0.039*
Disk tilt ratio	NA		0.692	0.004*
ß-zone PPA area/disk area	0.692	0.004*	NA	
**Treatment outcomes**				
Total No. of anti-VEGF injections	−0.025	0.930	−0.138	0.623
BCVA at the last visit	0.216	0.440	0.520	0.047*
BCVA changes during follow-up	0.101	0.719	0.050	0.859

*Statistically significant difference (*p* < 0.05, partial correlation analysis adjusted for age and sex). BCVA, best corrected visual acuity; CNV, choroidal neovascularization; PPA, peripapillary atrophy; NA, not available; and VEGF, vascular endothelial growth factor.

When we analyzed the optic disk features and changes during follow-up period (Table 3), we found that eyes with optic disk tilt had a significantly higher PDR at the initial (4.18 ± 3.16 vs. 1.78 ± 2.10, *P* < 0.001) and final presentation (6.03 ± 5.10 vs. 2.41 ± 2.88, *P* < 0.001). Regardless of the presence of optic disk tilt, the absolute degree of optic disk tilt (1.42 ± 0.27 to 1.59 ± 0.36) and PDR (3.28 ± 3.03 to 4.66 ± 4.72) were all increased during follow-up. However, the degrees of these changes were significantly larger in eyes with optic disk tilt (*P* = 0.036 and *P* = 0.013, respectively).

**TABLE 3:** Optic disk features and changes during follow-up according to the presence of optic disk tilt.

	Total eyes (*n* = 80)	Without tilted disk (*n* = 30)	With tilted disk (*n* = 50)	*P*-value
**Disk tilt ratio**				
Baseline	1.42 ± 0.27	1.16 ± 0.08	1.57 ± 0.22	<0.001*
Final	1.59 ± 0.36	1.29 ± 0.16	1.77 ± 0.32	<0.001*
Changes during follow-up	0.18 ± 0.18	0.13 ± 0.12	0.20 ± 0.20	0.036*
**ß-zone PPA area/disk area**				
Baseline	3.28 ± 3.03	1.78 ± 2.10	4.18 ± 3.16	<0.001*
Final	4.66 ± 4.72	2.41 ± 2.88	6.03 ± 5.10	<0.001*
Changes during follow-up	1.68 ± 2.11	0.63 ± 0.96	1.84 ± 2.46	0.013*

*Statistically significant difference (*p* < 0.05, Student’s t-test). PPA, peripapillary area.

**FIGURE 2:**
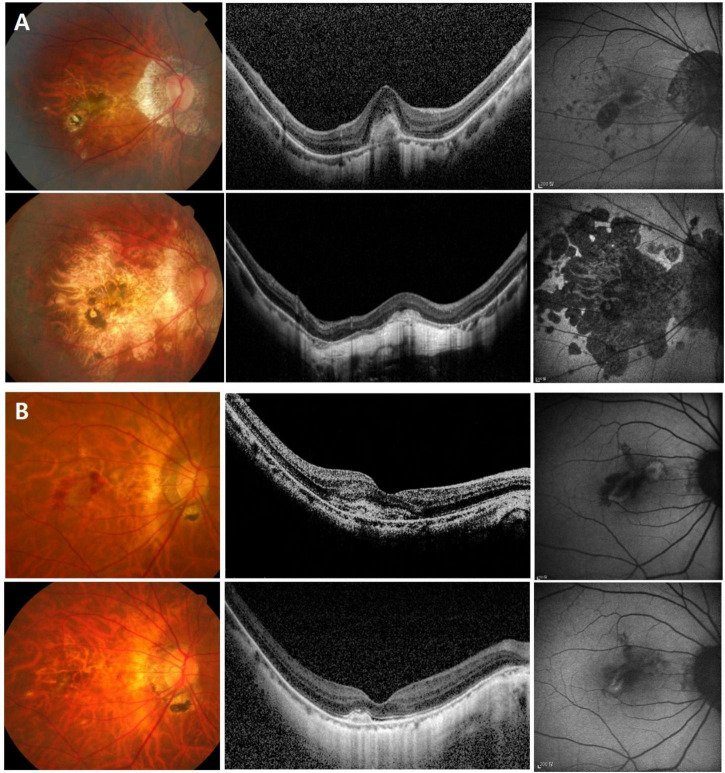
Color fundus photo, optical coherence tomography, and fundus autofluorescence images showing the greater progression of macular atrophy and worse visual outcome of subfoveal myopic choroidal neovascularization (CNV) in an eye with higher β-PPA-to-disc area ratio (PDR) and tilt ratio **(A)**, compared to an eye with lower PDR and tilt ratio **(B)**. In panel **(A)** a 65-year-old female patient with an axial length of 28.7 mm. At baseline (the upper row), visual acuity was 0.63 in Snellen with the high β-PPA-to-disc area ratio (PDR) (4.53), tilt ratio (1.55), and subfoveal myopic CNV. During six years of follow-up, the patients underwent two recurrences and received nine injections. At the last visit (the lower row), visual acuity was deteriorated to counting finger with the development of extensive CNV-related macular atrophy. In panel **(B)** a 65-year-old female patient with an axial length of 29.2 mm. At baseline (the upper row), visual acuity was 0.32 in Snellen with the low β-PPA-to-disc area ratio (PDR) (0.68), tilt ratio (1.32), and subfoveal CNV. During eleven years of follow-up, the patients underwent one recurrence episode and received three injections. At the last visit (the lower row), visual acuity was maintained at 0.6 in Snellen with little occurrence of macular atrophy.

To identify the risk factors for poor anatomical and functional outcomes, logistic regression and Cox proportional hazard models were conducted. In the multivariable analysis, larger baseline CNV size (OR = 8.461 [95% CI, 1.722–41.557], *P* = 0.009) and higher PDR (OR = 2.257 [95% CI, 1.454–3.505], *P* < 0.001) were found to be significant risk factors for the occurrence of macular atrophy at the last visit (Table 4). Meanwhile, older age (HR = 1.055 [95% CI, 1.012–1.100], *P* = 0.012) and higher PDR (HR = 1.174 [95% CI, 1.045–1.318], *P* = 0.007) were associated with poor final visual outcome (<20/60, Snellen mean; Table 5). Representative images demonstrate the worse anatomical and functional outcomes of subfoveal myopic CNV in eyes with higher PDR and tilt ratio (Figure 2).

**TABLE 4:** Baseline characteristics associated with the occurrence of macular atrophy in eyes with myopic choroidal neovascularization.

	Univariate analysis		Multivariable analysis	
	OR (95% CI)	*P*-value	OR (95% CI)	*P*-value
**Demographic factors**				
Age	1.075 (1.031–1.121)	0.001*		
Female sex	1.437 (0.441–4.682)	0.547		
Diabetes mellitus	1.001 (0.279–10.197)	0.990		
Hypertension	3.810 (0.903–16.068)	0.069		
**Ocular factors**				
Baseline BCVA	2.562 (1.005–6.531)	0.049*		
Axial length	0.961 (0.539–1.715)	0.893		
CNV size	6.034 (1.732–21.025)	0.005*	8.461 (1.722–41.557)	0.009*
Subfoveal choroidal thickness	0.990 (0.981–0.998)	0.020*		
Total No. of anti-VEGF injections	1.280 (0.964–1.910)	0.182		
Recurrence of CNV	1.611 (0.614–2.739)	0.156		
Disk tilt ratio	15.60 (0.181–134.04)	0.012*		
ß-zone PPA area/disk area	2.216 (1.471–3.338)	<0.001*	2.257 (1.454–3.505)	<0.001*

*Statistically significant (*p* < 0.05, Univariate and Multivariable Logistic regression model). HR, hazard ratio; BCVA, best corrected visual acuity; CNV, choroidal neovascularization; and PPA, peripapillary atrophy.

**TABLE 5:** Baseline characteristics associated with poor visual outcomes in eyes with myopic choroidal neovascularization.

	Univariate analysis		Multivariable analysis	
	HR (95% CI)	*P*-value	HR (95% CI)	*P*-value
**Demographic factors**				
Age	1.061 (1.030–1.094)	<0.001*	1.055 (1.012–1.100)	0.012*
Female sex	0.849 (0.429–1.681)	0.639		
Diabetes mellitus	0.757 (0.179–3.197)	0.705		
Hypertension	2.009 (1.004–4.021)	0.049*		
**Ocular factors**				
Baseline BCVA	2.077 (1.197–3.606)	0.009*		
Axial length	1.003 (0.659–1.528)	0.987		
CNV size	1.596 (1.103–2.311)	0.013*		
Subfoveal choroidal thickness	0.980 (0.965–0.996)	0.014*		
Total No. of anti-VEGF injections	1.009 (0.942–1.081)	0.803		
Recurrence of CNV	0.794 (0.379–1.662)	0.541		
Disk tilt ratio	3.709 (1.103–12.473)	0.034*		
ß-zone PPA area/disk area	1.225 (1.120–1.338)	<0.001*	1.174 (1.045–1.318)	0.007*

*Statistically significant (*p* < 0.05, Univariate and multivariable Cox proportional hazard regression model). HR, hazard ratio; BCVA, best corrected visual acuity; CNV, choroidal neovascularization; and PPA, peripapillary atrophy.

## Discussion

Our current study shows that myopic CNV in eyes with high PDR, indicating ß-zone PPA enlargement, was predisposed to worse anatomical and functional outcomes as well as worse baseline characteristics. Although PDR and tilt ratio were strongly correlated with each other in myopic CNV eyes, only PDR was an independent prognostic factor. To the best of our knowledge, this is the first study to investigate whether the PPA and disk tilt-related disk characteristics are associated with functional and structural outcomes of myopic CNV. This is a clinically significant observation as it highlights the importance of paying more attention to myopic CNV patients with myopic disk deformations, especially large ß-zone PPA. We expect PDR would be a useful parameter in the management of myopic CNV.

Although the underlying mechanisms of the association between enlargement of PPA and prognosis of myopic CNV are yet to be defined, we propose an explanation for our observation based on two aspects—structural deformation and hemodynamic change. First, PPA is associated with progressive structural deformation of the eyeball, which is related to well-known prognostic factors for myopic CNV such as old age, long axial length, and, more importantly, thin choroidal thickness (18–20). In a recent study (19), eyes with enlarged PPA were shown to have a thinner choroidal thickness and a higher risk for myopic maculopathy. Other studies (21, 22) also reported that the development or enlargement of PPA was a risk factor for the progression of myopic maculopathy and the progression from high myopia to pathologic myopia. Specifically, when the eyeball deformation is gradually aggravated with progressive eyeball elongation, the choroidal thickness in eyes with large PPA is more prone to further thinning and subsequent myopic chorioretinal atrophic changes. Further study is required to validate whether choroidal changes with elapse of time affect the prognosis in myopic CNV. The second possible explanation is that the differences in clinical course might be caused by perfusion insufficiency in association with peripapillary structural deformities. This speculation is supported by the study by Sung et al. (15), which reported the differences of retinal microvasculature according to the degree of optic nerve head deformation in highly myopic eyes. Other study (23) also suggested that significant choroidal changes lead to decreases in the choroidal blood flow and that ischemia-induced expression of growth factors caused by choroidal hypoperfusion is possibly related to the development and progression of myopic CNV. Further study is needed to elucidate the casual relationship between choroidal structural changes and choroidal perfusion.

In eyes with subfoveal myopic CNV, PDR and optic disk tilt were strongly associated. In addition, both of their degrees had increased during follow-up and these changes in optic disk morphology were more prominent in eyes with optic disk tilt. Interestingly, after adjusting for confounding factors and multivariable analysis, PDR was the most robust poor prognostic factor for subfoveal myopic CNV even in consideration with disk tilt. This difference might be due to the fact that PDR is a parameter comprising PPA and optic nerve head changes, (e.g., disk tilt), while the tilt ratio only represents optic nerve head morphologic changes. As an eyeball is elongated, nasalization of the optic nerve head and temporal stretching of sclera occur and lead to disk tilt and PPA, respectively, (24). Since PDR represents both changes, it would be a more potentially useful parameter for delineating degenerative myopic changes. In accordance with previous reports (6, 8–10), we also found that, respectively, larger baseline CNV and older age were significantly associated with occurrence of macular atrophy and poor final visual outcome.

One of the strengths of this study is that we focused only on subfoveal myopic CNV. Moreover, the follow-up duration and the number of patients were sufficient for the analysis of prognostic factors. Despite these strengths, the present study also has some limitations. First, due to the retrospective design of this study, the tilted optic disk could only be evaluated in two-dimensional fundus photos, which might not have precisely reflected the shape of the three-dimensional optic disk. Wakabayashi and Ikuno (23) showed that the increases in the ovality index did not represent the changes in posterior polar curvature but rather a shift of the temporal disk margin. Therefore, a three-dimensional assessment of the optic disk is required to elucidate the current study results further. Second, because the present study was conducted in a single university-affiliated hospital, its results might not be representative of the myopic population as a whole. Moreover, patients with early improvement may not have been included in this study because they were more likely to be lost to follow-up. Yet, the main goal of this study was to compare the clinical characteristics according to the presence of optic disk features, and considering that the limitations listed above were applicable to all of the study patients, the main results were not likely to have been significantly affected by the limitations.

In conclusion, after investigating the morphologic characteristics of the optic disk in eyes with subfoveal myopic CNV, we found that a high ß-zone PPA area/disk area ratio was an independent risk factor for poor anatomical and functional outcomes. Our findings suggest that myopic CNV in eyes with large ß-zone PPA should be given high priority for close observation.

## Data availability statement

The original contributions presented in this study are included in the article/supplementary material, further inquiries can be directed to the corresponding author.

## Ethics statement

The studies involving human participants were reviewed and approved by Institutional Review Board of Asan Medical Center (Seoul, South Korea; IRB No. 2020-1421). Written informed consent for participation was not required for this study in accordance with the national legislation and the institutional requirements.

## Author contributions

YK, JL, J-GK, and YY: study concept and design. YH and YK: data acquisition, management, analysis, interpretation, and manuscript draft. YK, HY, BM, JL, J-GK, and YY: revision, review, and approval. All authors contributed to the article and approved the submitted version.
